# Comparing the Air Abrasion Cutting Efficacy of Dentine Using a Fluoride-Containing Bioactive Glass versus an Alumina Abrasive: An* In Vitro* Study

**DOI:** 10.1155/2015/521901

**Published:** 2015-11-30

**Authors:** Melissa H. X. Tan, Robert G. Hill, Paul Anderson

**Affiliations:** Centre for Oral Growth and Development, Barts and The London School of Medicine and Dentistry, Unit of Dental Physical Sciences, Queen Mary University of London, Mile End Road, London E1 4NS, UK

## Abstract

Air abrasion as a caries removal technique is less aggressive than conventional techniques and is compatible for use with adhesive restorative materials. Alumina, while being currently the most common abrasive used for cutting, has controversial health and safety issues and no remineralisation properties. The alternative, a bioactive glass, 45S5, has the advantage of promoting hard tissue remineralisation. However, 45S5 is slow as a cutting abrasive and lacks fluoride in its formulation. The aim of this study was to compare the cutting efficacy of dentine using a customised fluoride-containing bioactive glass Na0SR (38–80 *μ*m) versus the conventional alumina abrasive (29 *μ*m) in an air abrasion set-up. Fluoride was incorporated into Na0SR to enhance its remineralisation properties while strontium was included to increase its radiopacity. Powder outflow rate was recorded prior to the cutting tests. Principal air abrasion cutting tests were carried out on pristine ivory dentine. The abrasion depths were quantified and compared using X-ray microtomography. Na0SR was found to create deeper cavities than alumina (*p* < 0.05) despite its lower powder outflow rate and predictably reduced hardness. The sharper edges of the Na0SR glass particles might improve the cutting efficiency. In conclusion, Na0SR was more efficacious than alumina for air abrasion cutting of dentine.

## 1. Introduction

*Broad Information on Topic*. The conventional method of treatment for dental caries would be to remove carious tooth tissue, followed by replacement using a restorative material. The most common method of caries removal today is via the dental air rotor drill. Despite its widespread use, some associated problems include dentinal sensitivity after use, high-pitched noises during use, thermal stimulation of the pulp tissue, bone-conducted vibration, and pressure within the tooth structure [1]. Some other methods that have been explored include caries removal using hand excavation [2], chemical agents like Carisolv [3], and air abrasion [4]. As dentistry moves towards minimally invasive treatment [5–9] and tooth-coloured adhesive materials gain favour over amalgam, the importance of creating cavities with well-defined walls and retentive undercuts has diminished.

As a caries removal technique, air abrasion refers to a nonrotary method of abrading a surface using a stream of high-speed abrasive particles generated from compressed air [10]. It possesses an end-cutting mode of action [11, 12] and creates saucer-shaped cavities with indistinct walls and margins [13]. As compared to rotary drilling, air abrasion does not cause vibrational forces on the tooth due to the minute sizes of the particles that come into contact with it [14]. As such, it is more comfortable and creates less stress on the tooth structure [15]. Particularly in the dentine region close to the pulp, where careful caries removal using the slow speed handpiece often proves uncomfortable due to the vibrations produced, slow-cutting air abrasion becomes a viable option. The high velocity particles propelled by the air stream also translate into less exertion required by the operator [14].

Commercially, aluminium oxide abrasives are used for cutting tooth tissue using air abrasion. However, issues of indiscriminate cutting of sound tooth structure [15] coupled with controversial health and safety issues [16, 17] make it a less ideal material. An alternative material is bioactive glass, which was originally designed as a biocompatible bone replacement material [18]. A bioactive glass abrasive, Sylc, is also commercially available but indicated for the purpose of tooth polishing. Some work has also showed potential for Sylc to have selective cutting properties [19, 20]. However, its cutting time can take 2-3 times longer than alumina, making it clinically impractical [4, 21]. Neither alumina nor Sylc possesses fluoride in its composition yet there is evidence that remineralisation in dentine can be achieved [22] with locally available fluoride ions [23].

While carrying out research on a new composition of fluoride incorporated bioactive glass, Farooq et al. [24] found that the hardness of bioactive glass could be increased by reducing its sodium content. This variability in hardness was then postulated to be useful in producing bioactive glass air abrasives. Moreover, their research demonstrated apatite formation within their series of bioactive glasses when it was placed in Tris buffer solution for 6–24 hours whereas Sylc produced smaller peaks of apatite in the same time. This study aims to study a glass with a similar base composition to Farooq et al.'s [24] and compare its efficacy against alumina in air abrasion cutting of dentine.

*Need for Study*. Studying the behaviour of a new bioactive glass abrasive, Na0SR, within the field of air abrasion cutting is needed. 

*Focus of Paper*. Determining the cutting efficacy of a customised fluoridated bioactive glass abrasive, Na0SR, against commercial alumina on dentine is the focus of the paper. 

*Null Hypothesis*. Na0SR and alumina cut dentine equally well. 

*Hypothesis*. Na0SR is not hard enough to cut dentine as efficiently as alumina. 

*Summary of Problem*. Caries removal via rotary instruments is quick but aggressive. It not only removes sound tissue very quickly, but also creates mechanical stresses within the tooth structure. Caries removal via other methods, that is, hand excavation or the use of chemical agents, for example, Carisolv, has proven to be slow and inconsistent. While air abrasion cutting of carious dental tissue has been increasingly studied in recent years, alumina abrasives still indiscriminately cut sound structure and do not possess remineralisation properties. Bioglass abrasives on the other hand have been shown to promote hard tissue remineralisation yet they abrade at a slower rate. It can therefore be postulated that a fluoridated bioactive glass abrasive that possesses apatite-forming abilities would have an edge over the two abrasive types mentioned above.

## 2. Materials and Methods

### 2.1. Bioactive Glass Abrasive Fabrication

Bioactive glass Na0SR was produced in the in-house lab using the melt-quench technique using the composition shown in Table 1. To produce glass of that molecular weight composition, 36.85 g of silicon dioxide, 14.35 g of phosphorus pentoxide, 131.89 g of strontium carbonate (all analytical grade products by Sigma-Aldrich, Gillingham, UK), and 6.25 g of strontium fluoride (Riedel-de Haën, Seelze, Germany) powder reagents (measured using weighing scale by Mettler PC 4400 Delta Range, Leicester, UK) were placed in a platinum crucible and heated in an electric furnace (Lenton, Derbyshire, UK) at 1540°C for 90 minutes. The viscous melted glass was then quenched by immediate pouring into a pail of deionised water at room temperature. The glass frit produced was immediately collected in a metal sieve and dried for at least 2 hours in a drying cabinet (LTE Scientific, Oldham, UK) at 65–80°C.

The dried glass frit was milled for 1 minute and 15 seconds using a milling machine (Gy-Ro Mill, Glen Creston, London, UK). This machine acts in a horizontal grinding motion and produces angular particles, ideal for air abrasion cutting. In order to select only abrasive particles between 38 and 80 microns in width, the ground powder was sieved using the Retsch VS 1000 sieve shaker (Retsch, Heidenheim, Germany) between two woven wire mesh analytical sieves (Endecotts Ltd., London, UK) of nominal apertures 38 and 80 microns for 30 minutes. The particle size distribution of the abrasives was visually examined under the Scanning Electron Microscope (SEM by Oxford Instruments, Abingdon, Oxfordshire, UK). Angular particles were observed but particles much smaller than 38 microns were noted to be present in masses. A second round of sieving was carried out for 30 minutes to remove the small particles.

**Figure 1:**
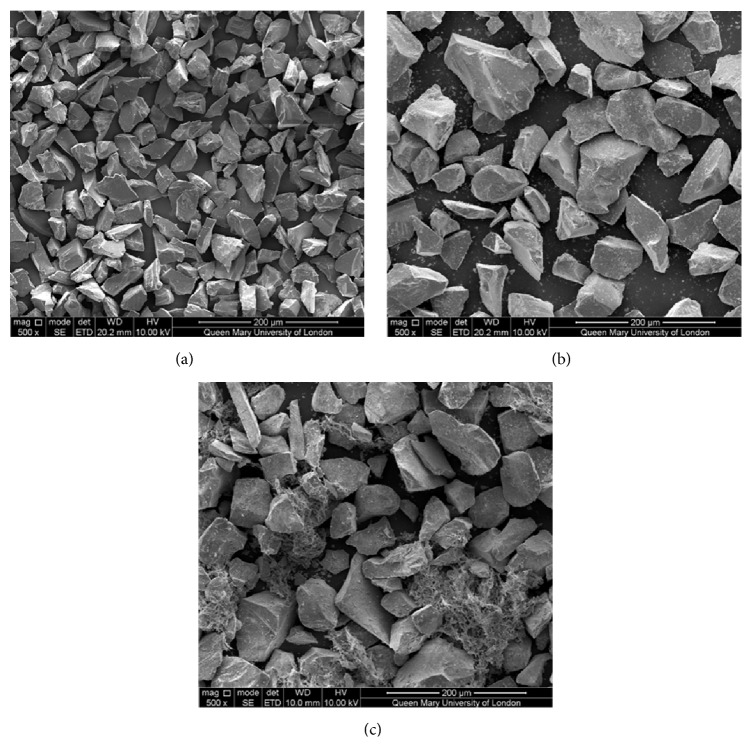
Scanning electron microscopy images of (a) alumina, adapted from Farooq et al. 2013 [24], (b) Na0SR, and (c) 45S5.

Rubber balls were placed in the 80- and 38-micron sieves in the first and second rounds of sieving, respectively. This was to discourage particle adherence and to encourage particle flow through the sieves. A second round of examination under the SEM confirmed a more even size distribution of angular particles within the targeted range of 38 to 80 microns in width (Figure 1).

**Figure 2:**
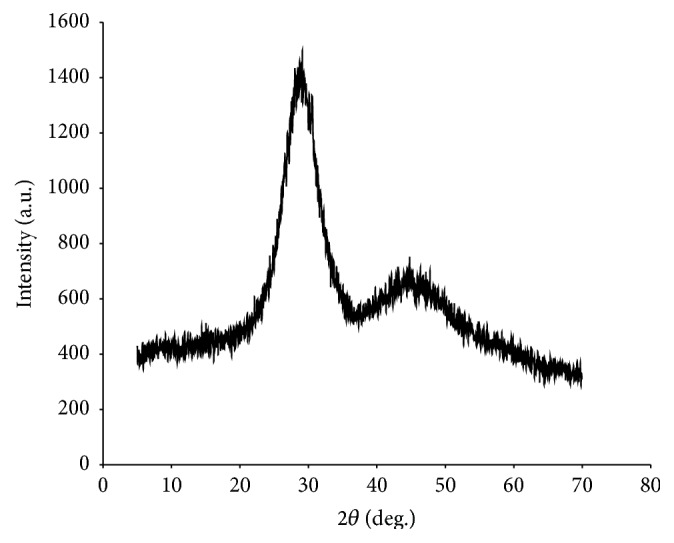
X-ray diffraction of one batch of Na0SR (#7) as an example.

Eight batches of glass with the same composition were produced. To check for homogeneity among the batches, glass characterisation was performed for each batch. Differential Scanning Calorimetry (DSC) was carried out (DSC, Stanton Redcroft DSC1500, Rheometric Scientific, Epsom, UK) to map out the behaviour of the glass through thermal changes. 0.05 g (weighing scale by Balance Technology, Wanstead, London, UK) of each glass batch was analysed to determine the glass transition temperature (*T*_g_). All eight batches of glass produced similar *T*_g_ values, which was indicative of the similarity in composition between them. The glass batches were also analysed using X-ray diffraction (XRD), a rapid analytical technique used for phase identification of a crystalline material. An amorphous material like glass should not have any phases identified. XRD analyses revealed that all eight batches of glass were generally amorphous with the exception of one narrow peak of crystallisation, indicative of a small but insignificant percentage of crystalline nature present (Figure 2). All eight batches of glass were then combined into one.

### 2.2. Powder Flow Tests

This was carried out to determine the consistency of the powder output of both abrasives. It was also to ensure that the output flow was smooth with no clogging.

**Figure 3:**
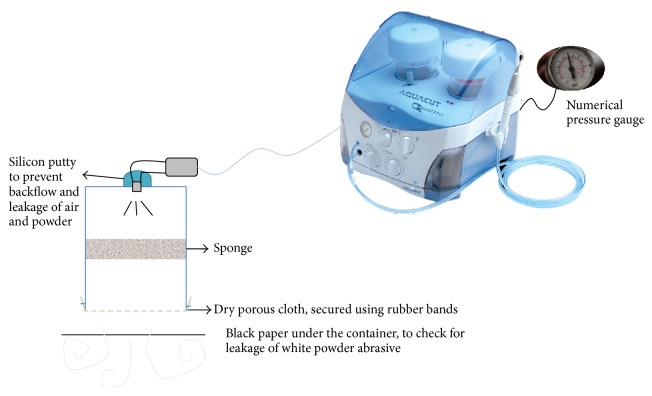
Air abrasion set-up of the abrasive powder outflow test.

The design of the set-up as shown in Figure 3 was similar to the system described by Banerjee et al. [25]. A commercially available air abrasion unit that had undergone recent maintenance and calibration was used (Aquacut Quattro, Twin-Chamber Dental Air Abrasion and Air Polishing Unit, Velopex International, Harlesden, London, UK). This machine dispenses abrasives using a vibration mechanism. Its silicon carbide nozzle tip, 0.6 mm in diameter, was directed through an airtight opening into a container sealed at the base with a dry, porous cloth. This cloth enabled air to escape but trapped the abrasive particles in. As both abrasives were white in colour, a contrasting black paper was kept beneath the cloth to ensure no particle leakage. A sponge was placed in the centre of the container to absorb the highly pressurised air jet from the nozzle and avoid fenestration of the cloth. The powder flow tests were carried out dry at a pressure of 552 kPa (80 PSI), with the powder flow setting kept at 2. The reservoirs containing the abrasive powders were maintained to be one-quarter-filled to half-filled throughout the experiment. The weight of each container before and after each run lasting for 30 seconds was measured (weighing scale by Mettler PC 4400 Delta Range, Leicester, UK). Five rounds were carried out for each abrasive.

### 2.3. Samples Preparation

In order to reduce variation between samples, ivory dentine slabs were used instead of extracted teeth. Ivory was obtained by the airport customs and later gifted to the department for research purposes. All samples were prepared from the same piece of ivory, taking care to ensure that the working surfaces were from the same plane. 14 flat slabs of ivory dentine, with working surfaces measuring 5 × 5 mm and a depth of at least 5 mm, were prepared using an annular diamond blade (Microslice 2 Precision Slicing Machine by Malvern Instruments, Malvern, England) within the confines of a fume cupboard (Astec Monair Astec Microflow, Bioquell UK Ltd., Hampshire, UK). The outer lining of cementum was removed and the samples were mounted onto acrylic resin blocks (Meadway cold cure rapid repair powder and liquid, MR Dental Supplies Ltd., Surrey, England, UK) to obtain a stable base. Polishing of the working surfaces was achieved using rotating silicon carbide paper wheels in incremental grit sizes to obtain a smooth, flat surface. To prevent desiccation of the dentine samples, they were stored in a moist, airtight container.

### 2.4. Air Abrasion Cutting Tests

14 samples of ivory dentine were used, with seven in each group of abrasives. Air abrasion tests were carried out using the same machine, Aquacut Quattro air abrasion machine (Velopex International, Harlesden, London, UK). It has a water spray feature coincident with and enveloping the particle stream. Two abrasive powders were compared, a commercial aluminium oxide abrasive (29 *μ*m) (Velopex International, UK), and the lab-fabricated fluoridated bioactive glass abrasive Na0SR (38–80 *μ*m). A 0.6 mm diameter nozzle tip was held stationary using a stand, at a distance of 1 mm and 90 degrees to the working surface of the dentine sample. The air abrasion was switched on for 10 seconds per round at a pressure of 552 kPa (80 PSI) and powder flow setting 2. Deionised water output was kept constant for this experiment. A rubber nozzle was used to funnel the water and the powder into a single stream. The nozzle was regularly checked to be centred and intact with no broken edges. The reservoirs holding the abrasives were kept between a quarter and half full throughout the experiment. Following each round of air abrasion treatment, a flush of deionised water over the working surface of each sample removed excess debris.

### 2.5. X-Ray Microtomography

Following the air abrasion procedure, the samples were scanned using X-ray microtomography on the MicroCT 40 Scanner (Scanco Medical, Switzerland). This is a nondestructive technique that characterises a material's microstructure in three dimensions at a micron level spatial resolution [26]. The specimen is mounted such that it can be stepped across a monochromatic beam and rotated about an axis normal to the beam. A detector measures the X-ray intensity and a line projection is calculated. A large number of line projections in multiple orientations can be collected to reconstruct a three-dimensional X-ray scan of the specimen [27].

An image of the plane through which the greatest cavity depth was observed for each sample was extracted from the three-dimensional scans using ImageJ program. Using a travelling microscope (Vickers Instruments, York, United Kingdom), physical measurements of the length of each slab as well as the diameter of the cavity were taken in the same plane as the extracted image. By calibrating the physical measurements with the length measurements on the image, the actual depths of the cavities were found.

## 3. Results

### 3.1. Powder Flow Tests (Figure 4)

The mean weight of alumina abrasive output was 6.75 g/min ± 6.9%, whereas the mean weight of Na0SR abrasive output was 5.92 g/min ± 3.6%. Assuming normal distribution, the two-tailed *t*-test demonstrated that the mean powder output of alumina versus Na0SR was significantly different at the 95% confidence interval (*p* = 0.023).

### 3.2. Cavity Depths Cut Using Air Abrasion (Figure 5)

The mean depth of cavity cut by alumina in ten seconds was 1.00 mm ± 8.4%, while Na0SR produced cavities of mean depth 1.19 mm ± 7.1%. Assuming normal distribution, the two-tailed *t*-test demonstrated that the mean cavity depths produced by Na0SR were nearly 0.2 mm deeper than alumina, significantly different at the 95% confidence interval (*p* = 0.001).

In conclusion, both the null hypothesis and hypothesis have been disproved. Na0SR abrasive was more efficacious at cutting dentine as compared to alumina despite its lower mean powder output.

## 4. Discussion

This study disproved the initial hypothesis. Instead of performing inferior to alumina in its cutting ability, the customised fluoridated bioactive glass abrasive Na0SR in this study produced significantly deeper cavities despite the lower powder outflow rate.

Bioactive glass is known to have a lower hardness as compared to alumina and it has also been consistently shown to cut at a slower rate than alumina [4, 19, 24]. Both Banerjee et al. [4] and Paolinelis et al. [19] used smaller particles of 45S5 Bioglass, 10–40 *μ*m and 25–32 *μ*m, respectively, similar in size to the alumina particles. The bioactive glass particles in this study were prepared as 38–80 *μ*m, in comparison, larger than the 29 *μ*m alumina particles used (Figure 1). It was modelled after the study by Farooq et al. [24], yet that study also demonstrated a lower cutting efficacy of bioactive glass. However, it might be useful to consider that the abrasives used in the study by Farooq et al. [24] appear to have multiple small particles within its distribution (observed from SEM images in the paper) and that might have affected the cutting ability or powder flow. The powder flow rate was not measured in that study.

**Figure 6:**
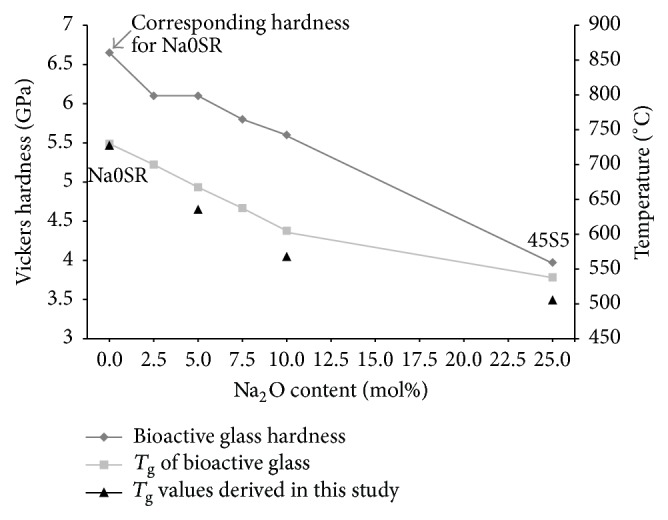
*T*_g_ and Vickers hardness values adapted from Farooq et al. 2013 [24], as comparison.

The composition of Na0SR was adapted from Farooq et al. [24], which showed that the *T*_g_ values of the glass corresponded to the changes in sodium composition and hardness of the glass. As sodium levels decreased, hardness levels increased. The *T*_g_ values derived in this study corresponded to the *T*_g_ values of the 0% sodium glass in that study (Figure 6). Its Vickers hardness is therefore postulated to be similar and close to 6.65 GPa. As compared to alumina which has a known Knoop Hardness Value (KHV) of 2100 [28, 29], approximately equivalent to 19.86 GPa in Vickers hardness, Na0SR is substantially softer. Both particles of alumina and Na0SR were angular in shape. To explain the observed favourable cutting outcome of Na0SR, we can postulate that it may be due to the nature of broken glass. Glass is brittle and produces sharp edges when broken. This increased surface area of sharp cutting edges upon impact could be the reason behind more efficacious tooth structure abrasion despite its lower volume output per minute.

**Figure 7:**
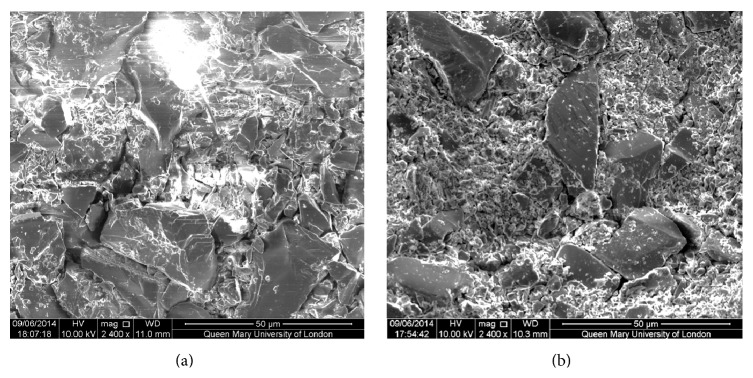
SEM image of the alumina (a) and Na0SR (b) remnant at the base of the cut cavities after air abrasion.

In the production of Na0SR, calcium was fully substituted by strontium from the zero-sodium composition in Farooq et al. [24] to increase the radiopacity of the abrasive for clearer XMT imaging. Due to the similarity in size and ionic charge of calcium and strontium, this substitution by molecular weight is not expected to change the structure [30, 31] or behaviour [32] of the glass. Farooq et al.'s [24] research demonstrated apatite formation within their series of bioactive glasses when it was placed in Tris buffer solution for 6–24 hours whereas Sylc produced smaller peaks of apatite within the same time frame. A comparison between the SEM images of the abrasives in their study and the SEM of Na0SR postoperatively (Figure 7) reveals the similarity of widespread small, broken down particles that would have an increased surface area for bioactivity to occur. Thus, Na0SR can be expected to behave in the same manner for apatite formation in Tris buffer solution.

The addition of fluorine, instead of substitution, ensured that the silicate network is not disrupted and the network connectivity remains unchanged [33]. Several studies have found that, by adding low amounts of fluorine to bioactive glass, fluorapatite is formed [34] and can be identified using MAS-NMR within six hours in Tris buffer [35]. This even occurs at a lower pH [36], which mimics an environment similar to an oral acid attack [37].

The important implication of this study is the emergence of an alternative abrasion cutting material, which not only has the ideal characteristics of being conservative and in line with minimally invasive dentistry, but also has likely remineralisation potential of hydroxyfluorapatite and uses a method with better patient acceptability due to its reduced vibrational forces, loud noises, and overall discomfort.

### 4.1. Limitations of Findings

**Figure 8:**
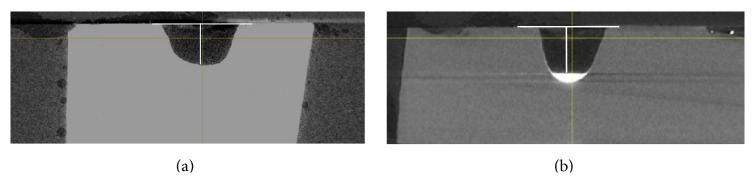
Samples of cavity cut by alumina ((a) with indistinguishable powder layer from dentine) and Na0SR ((b) with a very radiopaque powder layer due to the presence of strontium).

The jet of water spray on each sample was insufficient to remove the layer of powder compacted onto the base of the cavity. This layer is visible to the naked eye in every sample. However, due to a limitation of the X-ray microtomography machine, this layer can be differentiated clearly in some images but not in others (Figure 8). This has led to a systematic error in the collection of results, where an underestimation of the cavity depths is carried out for all samples by measuring the depths to the top surface of the powder layer, instead of to the actual cavity depth. Leaving the layer of powder in situ has its potential benefit and risk. A potential benefit would be that it could potentially act as a nucleus of minerals for remineralisation to occur. The plausible risk on the other hand would be an interference with the strength of bond between the restorative material and the tooth structure. This would need to be further studied. An initial examination was carried out via SEM images on the appearance of the powder layer after the procedure. Alumina had formed a layer comprising particles with similar sizes whereas Na0SR had a few larger particles dispersed and embedded within a bed of fine particles (Figure 7).

A second limitation would be a lack of comparison to 45S5 Bioglass in this study. The original plan was to compare the cutting efficacy of Na0SR to both 45S5 Bioglass and alumina. This could not be carried out due to the poor, inconsistent flow rate of the commercial 45S5 Bioglass (Sylc^©^, Aquacut Quattro, Velopex International, Harlesden, London, UK). Newly opened bottles of Sylc^©^ were revealed to have multiple, interspersed regions of web-like structure, which led to its irregular flow (Figure 1(c)). These structures have been suggested to appear similar to octacalcium phosphate [38], a precursor of hydroxyapatite that forms when in contact with moisture. The advantage of Na0SR over 45S5 Bioglass that can be postulated through this observation is its reduced sensitivity and reactivity to environmental moisture, which would translate to a longer shelf life.

Future research that would advance the field of air abrasion cutting using bioactive glasses would be to test the cutting efficacy of Na0SR on carious dentine as compared to sound dentine. Selective cutting of carious dentine over sound structure can be examined using a series of fluoridated glass compositions with varying sodium content and hardness.

## 5. Conclusion

Na0SR, a fluoridated bioactive glass, performed significantly better than alumina at air abrasion cutting of dentine. This is despite having a significantly lower abrasive particle output. Both were calculated at the 95% confidence level. Na0SR can therefore be considered as a plausible abrasive substitute for alumina in air abrasion cutting as it performs as well and has the potential added benefit of promoting remineralisation and hydroxyfluorapatite formation.

## Figures and Tables

**Figure 4:**
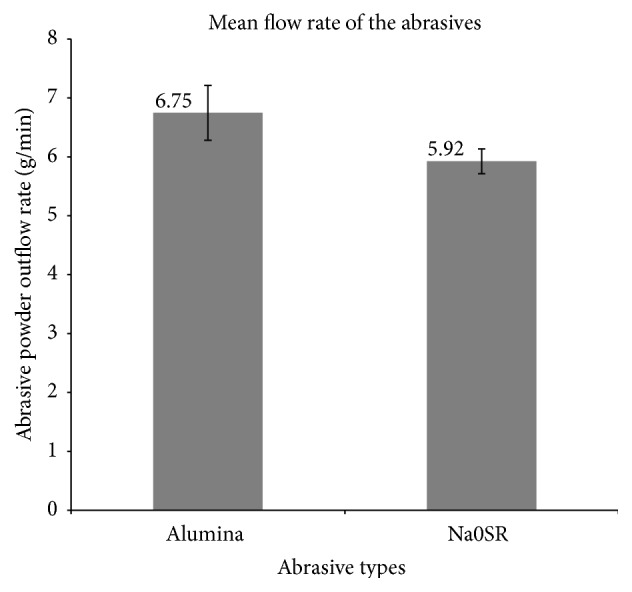
Comparison of mean flow rate between alumina and Na0SR abrasives.

**Figure 5:**
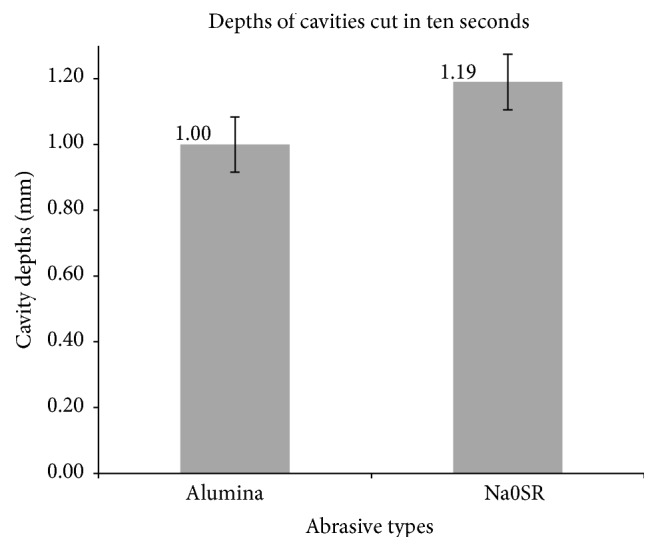
Comparison of cavity depths cut in 10 seconds between alumina and Na0SR abrasives.

**Table 1:** Composition of Na0SR by molecular weight percentage.

Na0SR composition	% by molecular weight
SiO_2_	37%
P_2_O_5_	6.1%
SrO	53.9%
SrF_2_	3%
